# Identification of Novel Markers That Demarcate the Nucleolus during Severe Stress and Chemotherapeutic Treatment

**DOI:** 10.1371/journal.pone.0080237

**Published:** 2013-11-06

**Authors:** Haitong Su, Mohamed Kodiha, Sunghoon Lee, Ursula Stochaj

**Affiliations:** McGill University, Department of Physiology, Montreal, Quebec, Canada; University of Campinas, Brazil

## Abstract

The nucleolus, the ribosomal factory of the cell, has emerged as a key player that regulates many aspects of cell biology. Several thousand proteins associate at least transiently with nucleoli, thereby generating a highly dynamic compartment with a protein profile which is sensitive to changes in cell physiology and pharmacological agents. Powerful tools that reliably demarcate the nucleoli are a prerequisite to measure their composition and activities. Previously, we developed quantitative methods to measure fluorescently labeled molecules in nucleoli. While these tools identify nucleoli under control and mild stress conditions, the accurate detection of nucleolar boundaries under harsh experimental conditions is complicated by the lack of appropriate markers for the nucleolar compartment. Using fluorescence microscopy we have now identified new marker proteins to detect nucleoli upon (a) severe stress and (b) drug treatments that trigger a pronounced reorganization of nucleoli. Our results demonstrate that nucleolin is an ideal marker to delimit nucleoli when cells are exposed to heat or oxidative stress. Furthermore, we show for the first time that cellular apoptosis susceptibility protein (CAS) and human antigen R protein (HuR) are excluded from nucleoli and can be employed to delimit these compartments under severe conditions that redistribute major nucleolar proteins. As proof-of-principle, we used these markers to demarcate nucleoli in cells treated with pharmacological compounds that disrupt the nucleolar organization. Furthermore, to gain new insights into the biology of the nucleolus, we applied our protocols and quantified stress- and drug-induced changes in nucleolar organization and function. Finally, we show that CAS, HuR and nucleolin not only identify nucleoli in optical sections, but are also suitable to demarcate the nucleolar border following 3D reconstruction. Taken together, our studies present novel marker proteins that delimit nucleoli with high confidence under a variety of experimental settings.

## Introduction

The nucleolus is a specialized compartment in the nucleus that serves as the site for ribosome biogenesis [1]. Nucleoli are assembled around chromosomal regions that contain tandem repeats of rDNA genes. These genes code for 45S pre-rRNA which is processed into 28S, 18S and 5.8S rRNAs [1], [2]. Processing of the 45S precursor relies on numerous factors and is a pre-requisite for the proper assembly of ribosomal subunits [3]. Aside from the assembly of ribosomal subunits, the nucleolus is implicated in a wide array of additional cellular functions. For instance, nucleoli regulate stress responses, cell cycle progression, apoptosis, telomerase activity, p53 stability, virus replication and bacterial infection [4], [5], [6], [7], [8]. At the structural level, the nucleolus is organized as a tripartite compartment that contains fibrillar centers, dense fibrillar components and the granular component [1], [2].

Since nucleoli participate in numerous biological processes, compromised nucleolar function is a hallmark of many human diseases and pathologies [9], [10]. For example, in mammalian cells the size and number of nucleoli reflect the need for ribosomal biogenesis, which is upregulated in many tumor cells. Thus, nucleoli are intimately linked to cancer, and nucleolar parameters, such as size and shape, have been used as diagnostic and prognostic tools in cancer therapy [11], [12]. Furthermore, nucleolar proteins are now primary targets for new anti-cancer drugs [13].

Proteomic studies for HeLa and other cells revealed that the nucleolus is composed of several thousand proteins [14]. Moreover, the nucleolus is highly dynamic, characterized by the continuous shuttling of proteins that move between the nucleolus and the surrounding nucleoplasm [15]. This dynamic nature allows nucleoli to respond and adjust rapidly to changes in cell physiology, especially when cells encounter stress [5], [16], [17].

Given the key role that the nucleolus plays in a multitude of cellular processes and its importance for human cell physiology, reliable tools are required to analyze the biological processes that take place in this compartment. Quantitative proteomics is a powerful approach to study the nucleolus. However, the fragile nature of nucleoli and the short residence time of many nucleolar shuttling proteins make it difficult for proteomics to capture the fast dynamic changes in this compartment. On the other hand, quantitative immunofluorescence is complicated by the lack of good markers that identify and demarcate the nucleolus unambiguously. This is even more challenging when nucleoli become fragmented, a process induced by stress, disease or treatment with pharmacological compounds [18], [19], [20], [21], [22], [23]. Such fragmentation can be associated with an extensive redistribution of nucleolar proteins, while components usually excluded from nucleoli may enter the compartment [24].

To begin to overcome these obstacles, we previously developed quantitative immunofluorescence protocols that employed markers which are excluded from the nucleolus, here referred to as “negative” nucleolar markers. In particular, our earlier studies relied on 4,6’-diamidino-2-phenylindole (DAPI) or RNA polymerase II (RNA Pol II) [21]. Although these techniques demarcated the nucleolar compartment properly under several experimental conditions, this does not apply universally to all settings tested. Therefore, additional markers are required to identify the nucleolus, especially for treatments that promote extensive reorganization of this compartment. To define the boundaries of the nucleolus under severe stress conditions, we have now evaluated the potential of three proteins to serve as markers. Specifically, we assessed nucleolin and two proteins that are concentrated in the nucleoplasm, but absent from nucleoli, cellular apoptosis susceptibility protein (CAS) and human antigen R protein (HuR).

Nucleolin is a multifunctional phosphoprotein that accounts for about 10% of the protein content in the nucleolus [25]. Like nucleolin, the importin-β family member CAS is involved in several cellular functions. As such, CAS serves as the nuclear exporter for importin-α, regulates p53-dependent transcription and controls apoptosis [26], [27]. Under normal growth conditions, CAS is present in the nucleoplasm [28], [29], [30], but excluded from nucleoli.

The RNA-binding protein HuR is synthesized in many mammalian cell types, where it associates with AU-rich element-containing mRNAs [31], [32], [33]. In unstressed cells, HuR is mostly nucleoplasmic and absent from nucleoli. Certain forms of stress, such as heat shock or oxidants, relocate a portion of HuR to cytoplasmic stress granules [34]. However, our previous work demonstrated that even upon stress a considerable amount of HuR remained in the nucleus, where it was excluded from nucleoli [35], [36]. The absence of CAS and HuR from nucleoli in unstressed cells suggests that these proteins provide suitable “negative” markers to identify the nucleolus under other growth conditions as well.

The research presented here evaluated nucleolin, CAS and HuR for their ability to delineate nucleoli under conditions that reorganize this compartment. To this end, we monitored the subcellular distribution of the candidates in cells exposed to heat shock or oxidative stress. Furthermore, protocols were designed to demarcate nucleoli upon treatment with agents that induce nucleolar fragmentation [16], [37]. For this purpose, we selected as representative chemotherapeutic drugs the transcription inhibitor actinomycin D and casein kinase II inhibitor 5,6-dichloro-1-β-D-ribofuranosyl benzimidazole (DRB) [38], [39], [40]. Our results demonstrate that nucleolin, CAS and HuR can be employed to identify nucleoli in different cell types under harsh experimental conditions that alter the organization and function of this subnuclear compartment. The protocols developed by us were applied to measure the impact of oxidative stress and pharmacological agents on nucleolar organization and function. To this end, we quantified how the different treatments affected (a) the abundance of B23/nucleophosmin and nucleolin, and (b) *de novo* RNA synthesis. Taken together, the research presented here provides guidance for the selection of markers that are suitable to demarcate nucleoli under different experimental settings.

## Materials and Methods

### Cell growth, stress exposure and drug treatment

HeLa and MCF7 cells were grown on poly-L-lysine coated cover slips to ∼70% confluency [28] and then exposed to different forms of stress. For severe heat shock, cells were incubated at 45.5°C for 1 hour. After heat exposure, cells were either fixed immediately or allowed to recover at 37°C for different periods of time. Control samples were kept at 37°C throughout the incubation period. Oxidative stress was generated by treating cells with 2 mM diethyl maleate (DEM) for 4 hours at 37°C [36]; the vehicle ethanol was added to controls. For experiments with pharmacological agents, cells were incubated at 37°C with 1, 10 or 100 nM actinomycin D for 6 hours [21] or 50 µM DRB for 4 hours. Actinomycin D and DRB were dissolved in DMSO and controls received DMSO only; DMSO was present at a final concentration of 0.1% (vol/vol) in all samples.

### Immunofluorescence

All steps were carried out at room temperature, essentially as described [21], [28]. In brief, following treatment, cells were rinsed with PBS, fixed with 3.7% formaldehyde/PBS for 20 min and washed with PBS. Cells were permeabilized with 0.1% Triton X-100 in PBS/2 mg/ml BSA/1 mM NaN_3_ for 5 min. Non-specific binding sites were blocked with PBS/0.05% Tween 20/5% fetal bovine serum, 1 mM NaN_3_ (blocking buffer; 1 hour), and samples were incubated overnight with primary antibodies against nucleolin (diluted 1∶1,000; Santa Cruz, sc-13057 ), CAS (1∶200; sc-1708), HuR (1∶1,000; sc-5261), HP1γ (1∶200; sc-365085), B23/nucleophosmin (1∶400; sc-271737), nucleostemin (1∶200; R&D Systems, AF1638). (Blocking buffer was used for the incubation with antibodies and all washing steps.) Specimens were washed and primary antibodies detected with affinity purified secondary antibodies raised in donkeys (Jackson ImmunoResearch): Alexa647-anti-goat (diluted 1∶200), Alexa488-anti-rabbit (1∶200), FITC-anti-rabbit (1∶200) or Cy3-anti-mouse (1∶500). After 2 hours, samples were washed and nuclei stained with 1 µg/ml DAPI (4,6′-diamidino-2-phenylindole). Cover slips were mounted and images were acquired for 0.7 µm slices using a Zeiss LSM510 inverted microscope equipped with a 63Χ oil-immersion objective (1.4 NA).

### Image processing and 3D reconstruction

Images were processed with Adobe Photoshop CS4; 3D reconstructions and surface rendering were performed with Imaris software (Bitplane). For 3D reconstruction and surface rendering, z-stacks were acquired for 0.3 µm slices with a Zeiss510 inverted microscope using a 63Χ oil-immersion objective (1.4 NA) and zoom 2.

### Specificity of antibodies for demarcation of nucleoli

Primary and secondary antibodies used to demarcate nucleoli were assessed by Western blotting and fluorescence microscopy. Crude cell extracts prepared for HeLa or MCF7 cells were probed side-by-side with primary antibodies and identical concentrations of isotype control IgG (Fig. S1) as previously described [41]. Primary antibodies against CAS, HuR, nucleolin or nucleostemin and fluorescently tagged secondary antibodies were further evaluated by fluorescence microscopy (Fig. S2). For each cell line, all of the images were acquired at the same settings of the microscope. In Fig. S2, channels refer to the signals observed for different wavelengths; channel 1, far red; channel 2, red; channel 3, green.

### Measurement of B23 and nucleolin in the nucleolus

Pixel intensities for B23 and nucleolin were measured in HeLa cells incubated with DEM or DRB. Nucleoli were demarcated with CAS or a combination of CAS and nucleolin as described in the figure legends. Pixel intensities/area were quantified for a minimum of three independent experiments. For every experiment, at least 30 cells were analyzed for each data point. Results for individual nucleoli are shown as average + SEM.

### Measurement of newly synthesized RNA in nucleoli

*De novo* synthesized RNA was labeled with 5-ethynyluridine (EU) and Alexa Fluor488 by click technology essentially as in reference [42]. In brief, samples were treated with vehicle, DEM, DRB or actinomycin D as described above. EU was present at a 0.5 mM final concentration during the last hour of the incubation period. Following treatment, cells were fixed and processed for immunostaining with antibodies against CAS and nucleolin. Experiments were repeated three times; in each experiment nucleolar pixel intensities were quantified for at least 30 cells/condition.

### Statistics

Fluorescent signals were measured in nucleoli of control and treated cells and normalized to controls. Each data point shows the average of at least three separate experiments plus SEM. Significant differences between control and treated samples were identified with Student’s *t-test*. Differences were considered significant for p-values < 0.05 and denoted with asterisks; *, p < 0.05; **, p < 0.01.

### Image analysis

**Figure 1:**
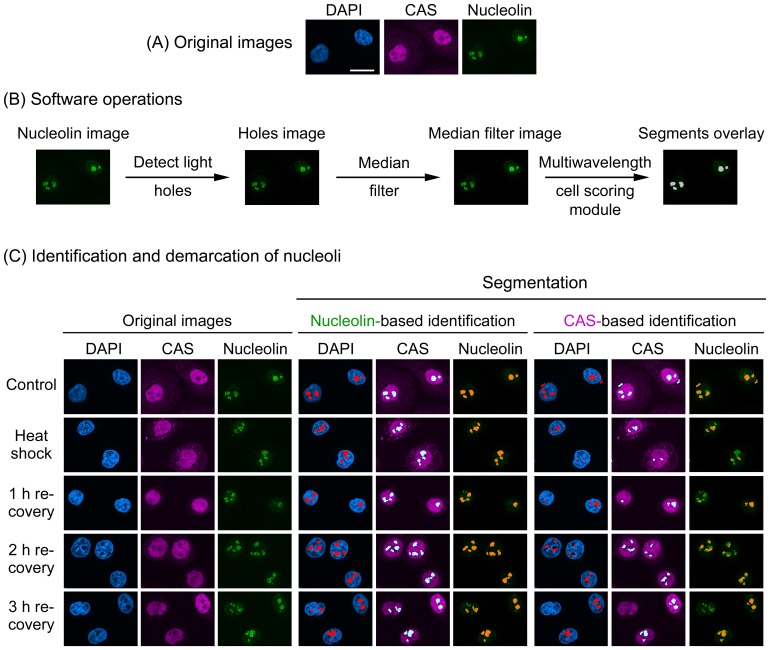
Nucleolin demarcates nucleoli upon heat shock and during the recovery from heat stress. (A) Original images for unstressed cells. (B) Software operations applied to detect nucleoli. The original image of the marker that demarcates the nucleolus (i.e. nucleolin) is subjected to a set of operations to define nucleoli and create nucleolar segments. In brief, the “detect light holes” filter identifies nucleoli based on the difference in fluorescence intensities between nucleoli and the surrounding nucleoplasm; this generates a “holes image”. The holes image is then passed through the “median filter” to reduce noise, generating the “median filter image”. The multiwavelength cell scoring module uses the median filter image as a template to produce segments that colocalize with nucleoli. The module accurately demarcates the boundaries of nucleoli on the basis of size constraints and intensity above local background [21]. (C) Demarcation of nucleoli in heat-stressed cells. HeLa cells were exposed to severe heat shock (1hour at 45.5°C), fixed immediately or after recovery at 37°C for the times indicated. Control samples were not heat-stressed. Fixed cells were processed for immunofluorescence staining with antibodies against nucleolin and CAS; nuclei were stained with DAPI. Confocal images were employed to identify and demarcate nucleoli with MetaXpress image analysis software. Segmentation and segments overlay panels depict the identification of nucleoli. Note that nucleolin delimited the nucleoli successfully under all conditions (Nucleolin-based identification). By contrast, CAS was not a suitable marker for heat-shocked cells until 3 hours recovery. Size bar is 20 µm.

Image analysis followed previously published protocols [21], [43] which were developed for MetaXpress software. In brief, the original image of the nucleolar marker protein is subjected to the “detect holes” filter to demarcate nucleoli. Depending on the marker, “detect light holes” or “detect dark holes” is applied. The “detect light holes” filter is used if the marker is concentrated in nucleoli; in this case the fluorescent intensities of nucleoli are higher than the surrounding nucleoplasm. Alternatively, if the marker concentration is low in the nucleolus (”negative” nucleolar marker), and nucleoli appear as dark holes, the “detect dark holes” filter is selected. As a result of this processing step, a “holes image” is generated, which is then processed with the median filter to reduce noise. The “median filter image” serves as a compartment image for which the multiwavelength cell scoring module produces segments that co-localize with nucleoli (Fig. 1A, B). Once generated, the segments are overlaid on the image to be analyzed and fluorescence intensities are quantified in nucleoli. Filters and software modules have previously been described in detail [21].

Image processing was also performed with ImageJ [44] for confocal images of immunostained nucleolin (see Supplemental figures). After input of the user-defined threshold, the software creates a threshold image. Based on the threshold image masks are generated. These masks are then overlaid with the original image to define and outline the segments.

## Results

**Nucleolin can demarcate nucleoli upon stress or actinomycin D treatment**

Heat shock is commonly applied to study the stress response, chaperone biology or other aspects of cell physiology. We have shown earlier that heat induces a relocation of the nucleolar proteins B23 and fibrillarin from the nucleolus to the nucleoplasm [21], preventing these proteins from serving as nucleolar markers during heat exposure. To overcome this obstacle, DAPI and RNA pol II were used either alone or in combination to identify nucleoli. Although the combination of DAPI and RNA pol II images improved the correct identification of nucleoli, this protocol requires a large number of processing steps and thus complicates image analyses.

**Figure 2:**
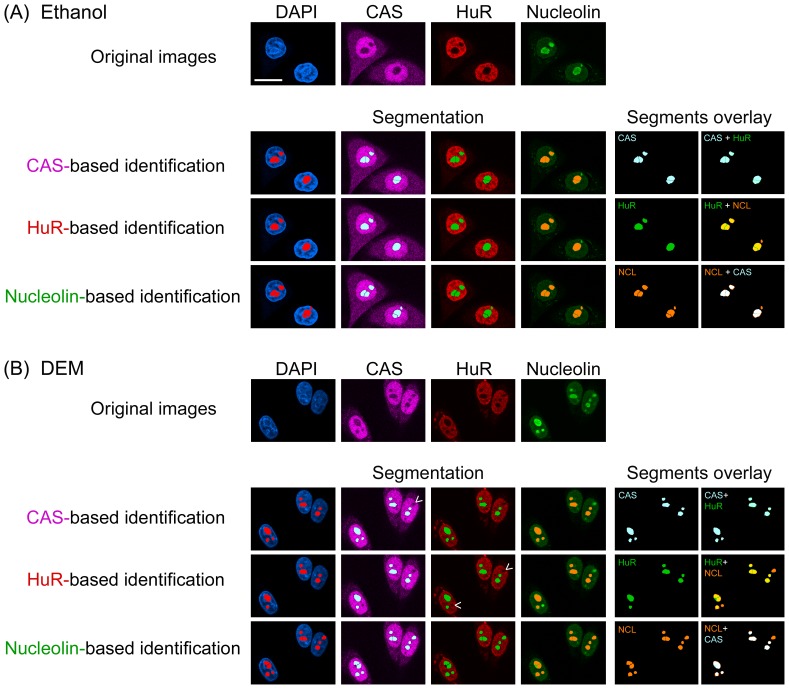
Nucleolin, CAS and HuR provide markers to demarcate nucleoli in oxidant-treated HeLa cells. HeLa cells were incubated with (A) the vehicle ethanol or (B) the oxidant DEM as described [36]. Immunostaining of CAS, HuR and nucleolin was carried out as for Fig. 1, and nucleoli were identified based on these markers. Segments generated for CAS, HuR or nucleolin and their overlays are shown on the right side. Note that nucleolin was superior to demarcate nucleoli in DEM-treated cells. While CAS and HuR were useful to delineate nucleoli, this occurred with reduced accuracy. Non-identified nucleoli are marked with arrowheads; size bar is 20 µm.

On the basis of these previous observations, it was our objective to streamline the processing without compromising the accuracy of nucleolar demarcation. To achieve this, we examined the potential of new candidates to serve as a nucleolar marker under heat stress and other conditions. Specifically, we assessed the candidates with our previously published protocols that had been developed for MetaXpress software [21]. A first set of experiments evaluated nucleolin in control and heat-shocked cells, either immediately upon stress exposure or after recovery for 1, 2 or 3 hours (Fig. 1C). As expected, the protein was concentrated in the nucleolus of control samples. Moreover, a considerable amount of nucleolin remained associated with nucleoli during heat shock and throughout the recovery period (Fig. 1C). This property distinguished nucleolin from other nucleolar proteins, such as B23 and fibrillarin, which both exit the nucleolus of heat-stressed cells [21]. A similar picture emerged when cells were exposed to oxidative stress, as nucleolin remained associated with the nucleolus of DEM-treated cells (Fig. 2). This suggested nucleolin can demarcate nucleoli (i) upon heat shock, (ii) during recovery from heat and (iii) after oxidant exposure. Indeed, applying the multiwavelength cell scoring module on the nucleolin image accurately detected and demarcated nucleoli for all of these conditions (Fig. 1C, Fig. 2; nucleolin-based identification).

As nucleolin is particularly concentrated in the dense fibrillar component (reviewed in [45]), it was important to confirm that it is suitable to detect other parts of the nucleolus as well. To this end, we compared the distribution of nucleolin and nucleostemin, a protein enriched in the granular component [46], [47]. In HeLa and MCF7 cells, nucleostemin was present in the area occupied by nucleolin (Fig. S3). The same was observed for B23/nucleophosmin, which is another resident of the granular component (see below). Thus, nucleolin can be employed to detect several nucleolar subcompartments.

The nucleolus is surrounded by heterochromatin which is highly condensed (reviewed in [48]), and it was possible that a heterochromatin marker could serve for compartment identification. We therefore tested HP1γ, a protein that associates preferentially with heterochromatin [49], for its ability to demarcate the nucleolus (Fig. S4). While nucleoli appeared as dark holes in HP1γ images, the border between nucleoli and the surrounding nucleoplasm was not well defined (compare HP1γ to CAS or nucleolin in Fig. S4). Hence, the markers described by us were superior to HP1γ for nucleolar identification.

**Figure 3:**
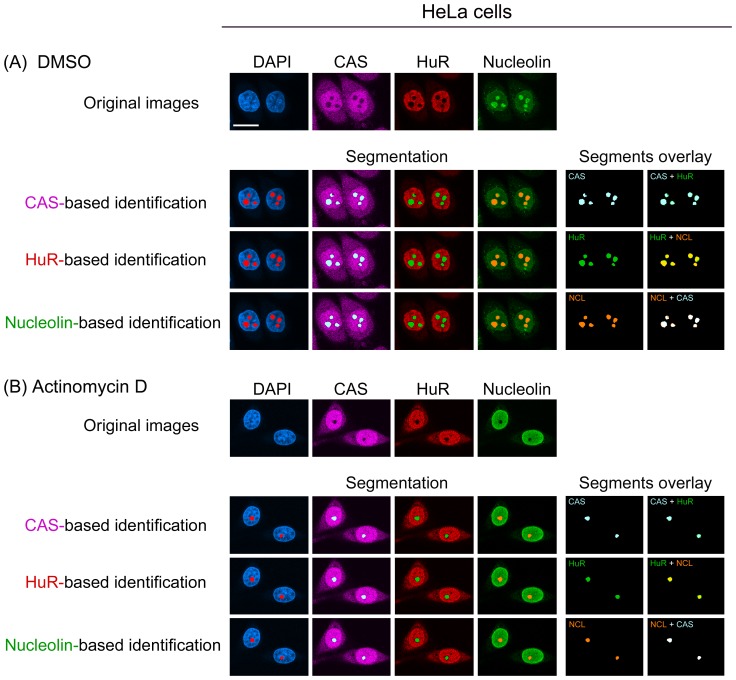
Nucleolin, CAS and HuR identify the nucleolus in HeLa cells treated with actinomycin D. HeLa cells were incubated with (A) the solvent DMSO or (B) 100 nM actinomycin D according to ref. [21]. Following treatment, samples were processed as in Fig. 2, and confocal images were used to identify nucleoli. Comparison of the segments and their overlay reveals that in control and actinomycin D-treated cells, all of the three proteins served as appropriate markers for nucleoli. Size bar is 20 µm.

While the results for nucleolar identification in heat and oxidant-stressed cells were encouraging, experimental settings that lead to nucleolar disassembly present a more difficult scenario. For instance, inhibition of RNA Pol I-mediated transcription by actinomycin D is one of the treatments that fragment the nucleolus [19]. As an inhibitor of RNA Pol I and ribosome biogenesis, actinomycin D has become an indispensible tool to study the biological properties of the nucleolus. Actinomycin D treatment leads to the redistribution of many nucleolar proteins [19] and thereby complicates the demarcation of nucleoli. Consistent with these observations, actinomycin D completely relocated nucleolin within the nucleus (Fig. 3).

**Figure 4:**
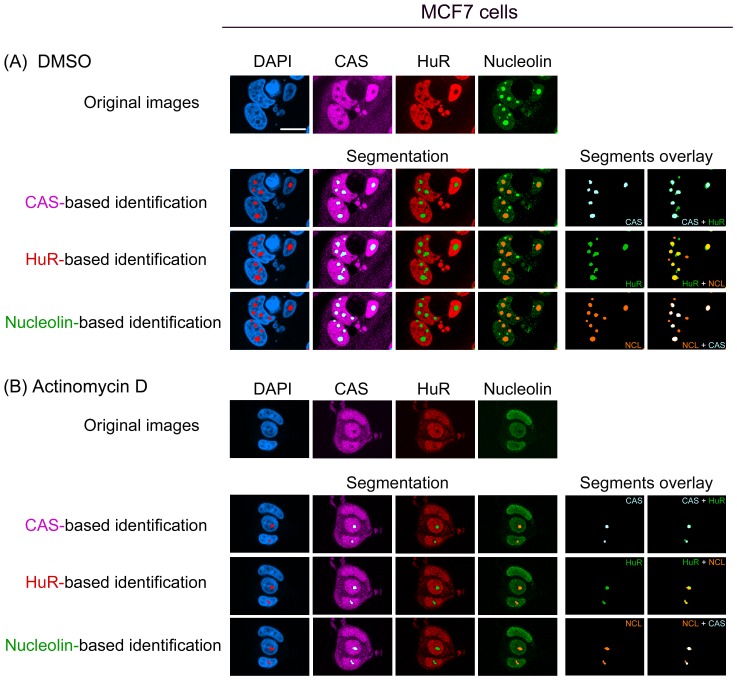
Nucleolin, CAS and HuR are suitable to detect nucleoli in actinomycin D-treated MCF7 cells. MCF7 cells were treated with (A) DMSO or (B) 100 nM actinomycin D. Cells and images were processed as described for Fig. 2. Size bar: 20 µm.

Interestingly, following the incubation with 100 nM actinomycin D, we detected dark holes for nucleolin inside the nucleus (Fig. 3B). Results described for CAS (see below) are consistent with the idea that the dark holes obtained for nucleolin staining indeed represent nucleoli. When the “detect dark holes” filter was used on the nucleolin image, we were able to identify this compartment in actinomycin D-treated HeLa cells (Fig. 3B, nucleolin-based identification). Since the impact of pharmacological agents on nucleoli is particularly relevant to cancer therapy, marker proteins were further evaluated in breast cancer cells. Indeed, this confirmed CAS, nucleolin and HuR as appropriate references for the identification of nucleoli in MCF7 cells (Fig. 4 and below).

At 100 nM (Fig. 3, 4), actinomycin D inhibits RNA Pol I, but may also affect RNA Pol II and thereby impinge on the overall organization of the nucleus [50]. To determine whether our detection method was also valid for lower drug concentrations, cells were incubated with 1 nM or 10 nM actinomycin D and nucleoli identified with the “detect dark holes” filter. Fig. S5 shows that under these conditions, CAS, HuR and nucleolin were suitable markers to delimit the nucleolus.

As the nucleolar organization is intimately linked to ribosome biogenesis and thus cell proliferation, it provides an excellent read-out to examine the mode of action for anti-cancer drugs [19], [40]. Methods to assess the state of nucleoli in drug-treated cells are therefore essential to improve chemotherapy. DRB is a pharmacological agent that leads to nucleolar fragmentation [37]; this treatment presents a challenge, because nucleolar remnants have to be identified.

**Figure 5:**
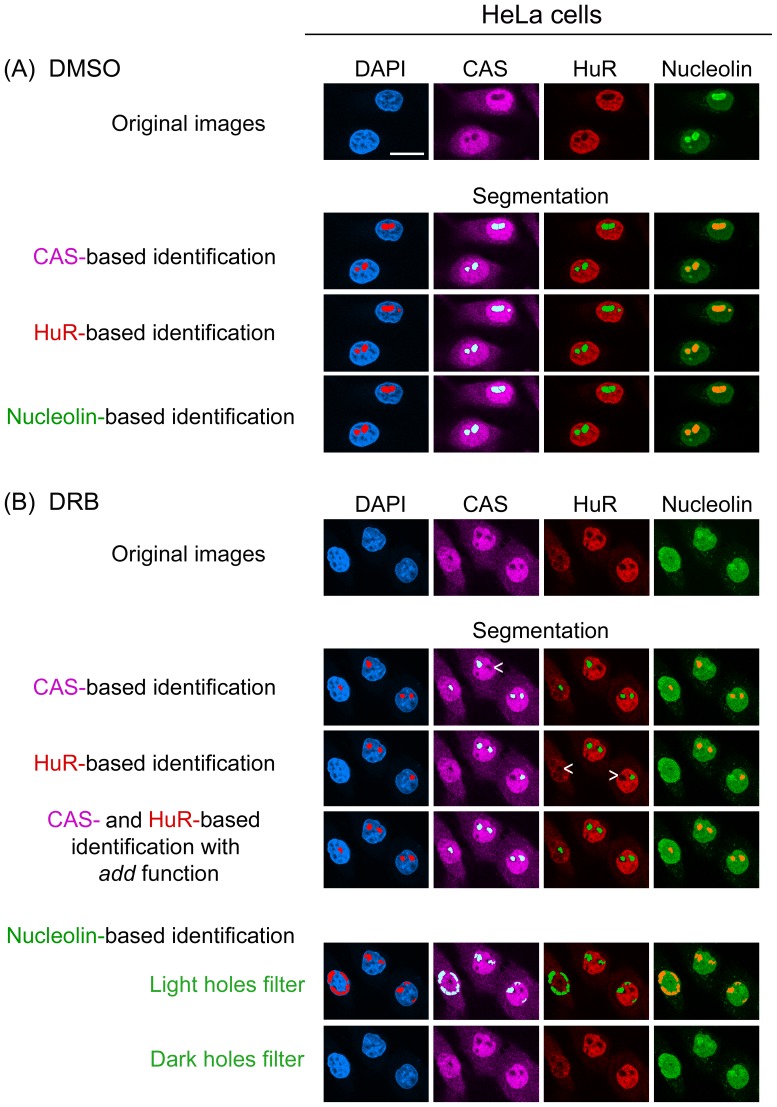
CAS and HuR, but not nucleolin, delimit nucleoli in DRB-treated HeLa cells. Cells were incubated with (A) DMSO or (B) DRB essentially as described [21] and processed as in Fig. 2. Individually, the marker proteins CAS and HuR detected nucleoli upon DRB incubation, although some nucleoli were missed (indicated by arrow heads). The identification of nucleoli was improved by combining the information from CAS and HuR images with the *add* function [21]. Nucleolin was redistributed by DRB throughout the nucleoplasm. Based on the nucleolin image, neither the “detect light holes” nor “detect dark holes” filter could identify nucleoli. Size bar is 20 µm.

**Figure 6:**
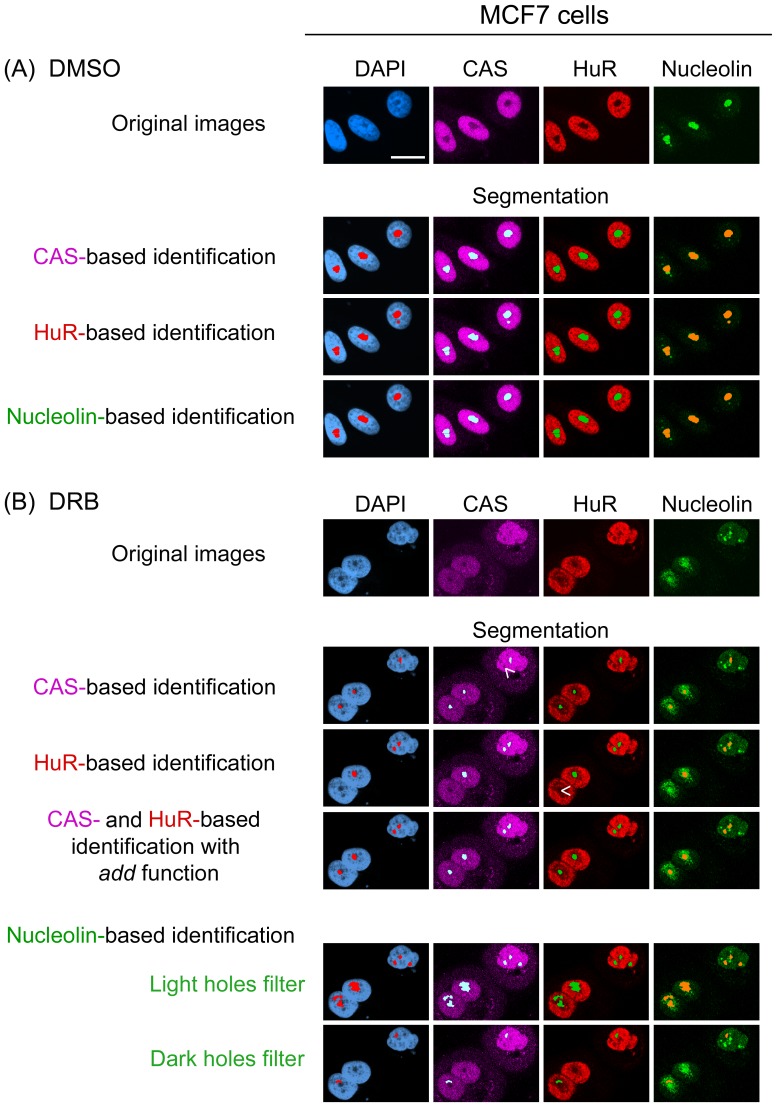
Demarcation of nucleoli in DRB-treated MCF7 cells. MCF7 cells were exposed to DRB and immunostained as described for Fig. 5. Segmentation is shown for control and stress conditions. When CAS and HuR were used as individual markers, some nucleoli were missed (arrow heads). However, these nucleoli were identified properly when CAS and HuR were combined to define compartments. Size bar is 20 µm.

Since nucleolin properly demarcated the nucleolus under several conditions (Fig. 1–4), we investigated its performance in DRB-incubated HeLa and MCF7 cells. However, upon DRB treatment nucleolin redistributed throughout the nucleus in many cells, and the “detect light holes” filter generated segments that appeared random (Fig. 5, 6; nucleolin-based identification). Hence, the compartment identification based on the nucleolin image was not reliable. At the same time, attempts to detect nucleoli with the “detect dark holes" filter failed (Fig. 5, 6). Consequently, alternative markers were required that delimit the nucleolar compartment when it is reorganized by harsh treatments, as exemplified by the chemotherapeutic agent DRB.

### CAS delimits nucleoli upon treatment with DRB, actinomycin D, and the oxidant DEM

The assessment of nucleolar damage induced by severe environmental changes or pharmacological agents requires markers that define nucleoli when they undergo fragmentation. As this process can redistribute many nucleolar proteins, we explored an alternative approach that relies on non-nucleolar proteins. To this end, we examined whether proteins that are excluded from nucleoli under normal growth conditions can identify the compartment by providing a “negative” marker. We hypothesized that such proteins could remain excluded from nucleoli upon stress and drug treatment. In this scenario, nucleoli are detected and demarcated with the dark holes filter. Importantly, boundaries of the nucleolar compartment can be defined even if nucleolar proteins redistribute.

To develop this method, we evaluated CAS and HuR as potential “negative” markers that are absent from the nucleolus. The rationale for choosing CAS and HuR is their residence in the nucleoplasm, but exclusion from nucleoli (Fig. 1, 2, 3, 4, 5, 6; control; Ethanol, DMSO). Accordingly, upon fluorescent staining of CAS or HuR, nucleoli were identified as “black holes” inside the nucleus. Fig. 1, 2, 3, 4, 5, 6 demonstrate that the dark holes in the nucleus obtained for CAS and HuR staining indeed represented nucleoli, because they co-localized with nucleolin.

We further assessed the potential of CAS as a reference in DRB-treated cells (Fig. 5, 6). In DMSO controls, CAS was concentrated in the nucleus, but excluded from nucleoli. Importantly, upon DRB incubation nucleoli continued to appear as black holes in the CAS image (Fig. 5B, 6B). Application of the multiwavelength cell scoring module on CAS-derived median filter images demarcated nucleoli in control and DRB-incubated cells (Fig. 5, 6, segmentation). However, when compared to DMSO controls (Fig. 5A, 6A), CAS-based nucleolar identification was less precise for DRB-treated cells and a small number of nucleoli was missed (Fig. 5B, 6B, arrowheads).

Given that CAS demarcates nucleoli in DRB-treated cells, we examined whether this also pertains to severe heat shock, oxidative stress and actinomycin D. Following heat shock and during the first hour of recovery, CAS redistributed within the nucleus and a considerable amount was detected in nucleoli. To the best of our knowledge, this is the first time that CAS was detected in nucleoli. Although we could not rely on CAS to delineate nucleoli during these time points, CAS began to exit the nucleolus at later stages of recovery, and black holes were reinstated in the CAS image. Hence, the use of CAS as nucleolar marker was partially successful after 2 and 3 hours recovery (Fig. 1). Unlike heat shock, oxidative stress did not induce a profound relocation of CAS within the nucleus, since the protein remained concentrated in the nucleoplasm and excluded from nucleoli. Although CAS did not outperform nucleolin, it delimited successfully nucleoli in oxidant-treated cells (Fig. 2, compare CAS-based to nucleolin-based identification. Note that in this representative image only one nucleolus was missed when CAS served as marker.). Similarly, CAS delimited successfully nucleoli in actinomycin D-treated cells (Fig. 3B, 4B).

Taken together, CAS provides a suitable reference for nucleolar demarcation, because the protein is absent from the compartment during DRB and actinomycin D treatment or exposure to oxidative stress. By contrast, CAS is not appropriate to define nucleolar borders in heat-shocked cells.

### HuR reliably delimits nucleoli upon DRB, actinomycin D or oxidant treatment, but not for heat shock

In DRB-incubated cells the CAS-based identification detected nucleoli, but a small number was missed under these conditions. Therefore, we compared the performance of HuR as an alternative reference by simultaneous staining of CAS, HuR and nucleolin (Fig. 5, 6). Following exposure to DMSO or DRB, HuR remained predominantly in the nucleoplasm and demarcated nucleoli. However, the precision was lower in DRB-treated cells when compared to DMSO controls (Fig. 5, 6; HuR- based identification, arrowheads). In addition to DRB, HuR properly delimited nucleoli in cells incubated with actinomycin D (Fig. 3B, 4B). A comparison between different markers and segments overlay showed that for actinomycin D results were comparable for HuR, CAS and nucleolin-based identification.

**Figure 7:**
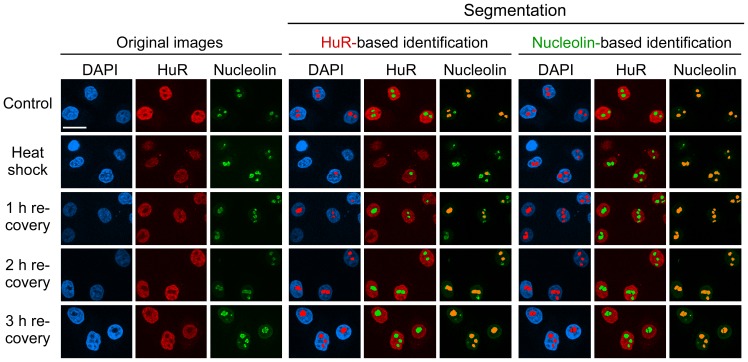
HuR is not a suitable nucleolar marker in heat stressed cells. HeLa cells were heat-shocked and immunostained for HuR and nucleolin. Nucleolin outperformed HuR for the compartment identification during heat shock and at 1 hour of recovery. After longer periods of recovery (2 and 3 hours), however, HuR identified nucleoli properly. Size bar: 20 µm.

When cells were exposed to the oxidant DEM, a portion of HuR relocated to cytoplasmic stress granules. Nevertheless, nucleoli could still be demarcated based on the HuR distribution (Fig. 2), although CAS and nucleolin provided more accurate identification in DEM-treated cells. In contrast to oxidative stress, HuR entered the nucleolar compartment upon heat shock, where some of the protein remained during the first hour of recovery (Fig. 7). Following this nucleolar association, HuR exited the nucleolus at later time points of recovery. For that reason, attempts to detect nucleoli based on HuR distribution failed in heat-shocked cells, but were successful after 2 and 3 hours of recovery. Taken together, HuR delimited nucleoli effectively in cells treated with DRB, actinomycin D or DEM, but did not perform reliably in heat-shocked cells.

### Combining the information from CAS and HuR distribution improves the accuracy of nucleolar detection in DRB-treated cells

As described in the previous sections, CAS and HuR defined the nucleolar boundaries upon DRB treatment when applied individually; however, it was desirable to improve the accuracy of detection. To achieve this, we combined the pixel intensities for CAS and HuR to generate a new image. This new image (Fig. 5, 6) was produced with the arithmetic *add* function [21] and greatly enhanced the difference between fluorescence signals in nucleoli and the surrounding nucleoplasm. The add image was then processed with the “detect dark holes” filter to delimit nucleoli. As depicted in Fig. 5, 6, the *add* strategy increased the number of nucleoli that were demarcated accurately. Thus, the combination of two markers absent from nucleoli improved the identification when compared to protocols that employed CAS or HuR individually. While we have combined the information from DAPI and Pol II-images in previous applications [21], it should be emphasized that some chemotherapeutic drugs affected the DAPI-staining (see for example Fig. 5B), and DAPI was no longer a dependable reference to delimit nucleoli. The combination of CAS and HuR images circumvented this problem and therefore provides a more reliable option for nucleolar identification.

### B23 and nucleolin have different sensitivities to DEM, but similar responses to DRB

**Figure 8:**
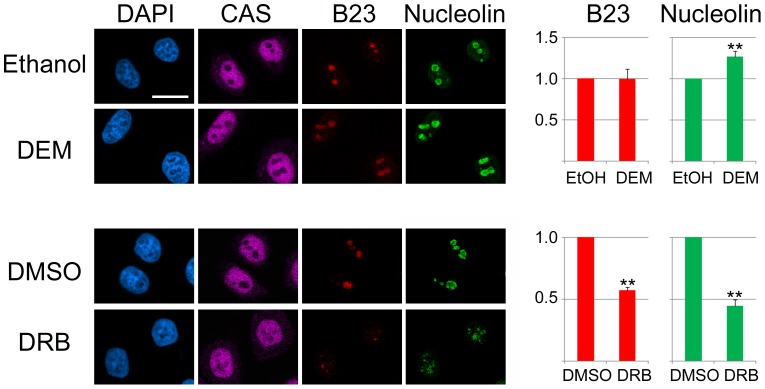
DEM and DRB change the protein composition of nucleoli. HeLa cells were treated with vehicle, DEM or DRB as detailed in Materials and Methods. Nucleolar pixel intensities for B23 and nucleolin were quantified for the same nucleoli. CAS and nucleolin were combined as markers for samples treated with ethanol or DEM. CAS alone provided a reference for cells incubated with DMSO or DRB. Multiple independent experiments were analyzed for DEM (4 data sets) and DRB (3 data sets). Segmentation images were inspected visually to eliminate misidentified nucleoli. Nucleolar fluorescence was then measured for at least 30 cells for each condition and experiment. Results are normalized to controls and depicted as average +SEM; *, p < 0.05 or **, p < 0.01. Size bar: 20 µm.

B23 and nucleolin are essential for nucleolar functions; in particular, both proteins are required for ribosomal biogenesis (reviewed in [25], [51], [52]). Our previous and current results show that B23 [21] and nucleolin exhibit distinct sensitivities to heat stress, and the same may apply to other treatments. Here, we applied our tools to further characterize the sensitivities of B23 and nucleolin to stress or pharmacological agents. In Fig. 8, the effect of DEM and DRB was analyzed in HeLa cells. To this end, pixel intensities were quantified for B23 and nucleolin in the *same* nucleoli. While DEM treatment had no effect on the abundance of B23 in nucleoli, a small but significant increase was observed for nucleolin. In contrast, DRB reduced significantly the concentration of both proteins in nucleoli; a similar drop in nucleolar signals was observed for B23 and nucleolin.

### DEM, actinomycin D and DRB inhibit transcription in the nucleolus

**Figure 9:**
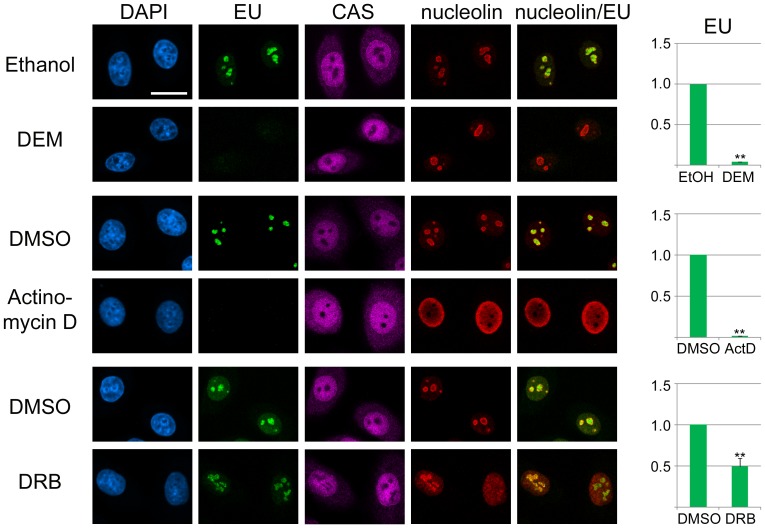
DEM, actinomycin D and DRB inhibit *de novo* RNA synthesis in nucleoli. HeLa cells were incubated with vehicle, DEM, actinomycin D or DRB as described in Materials and Methods. 5*-* **Ethynyluridin**e (EU) was present during the last hour of the incubation period, and cells were processed for the detection of EU, CAS and nucleolin [42]. Three independent experiments were performed for each compound. Misidentified nucleoli were removed from the analysis by visual inspection. Nucleoli of at least 30 cells were quantified for each condition and experiment. Data were normalized to controls and shown as average +SEM. Significant differences are indicated by *, p < 0.05 or **, p < 0.01. Size bar: 20 µm.

Since DEM, actinomycin D and DRB caused a re-organization of nucleoli, it was important to determine the possible consequences at the functional level. To address this point, *de novo* synthesized RNA was labeled during the last hour of the incubation period and detected by fluorescence microscopy, side-by-side with CAS and nucleolin. Nucleoli were demarcated based on CAS and nucleolin images in cells incubated with DEM or actinomycin D; CAS alone served as a marker in DRB-treated cells. A significant reduction in RNA synthesis was observed for all compounds tested, but the inhibition was especially pronounced with actinomycin D (Fig. 9). Hence it can be concluded that treatments changing the nucleolar organization were accompanied by a loss of nucleolar function.

### CAS, HuR and nucleolin demarcate nucleoli for 3D analyses

**Figure 10:**
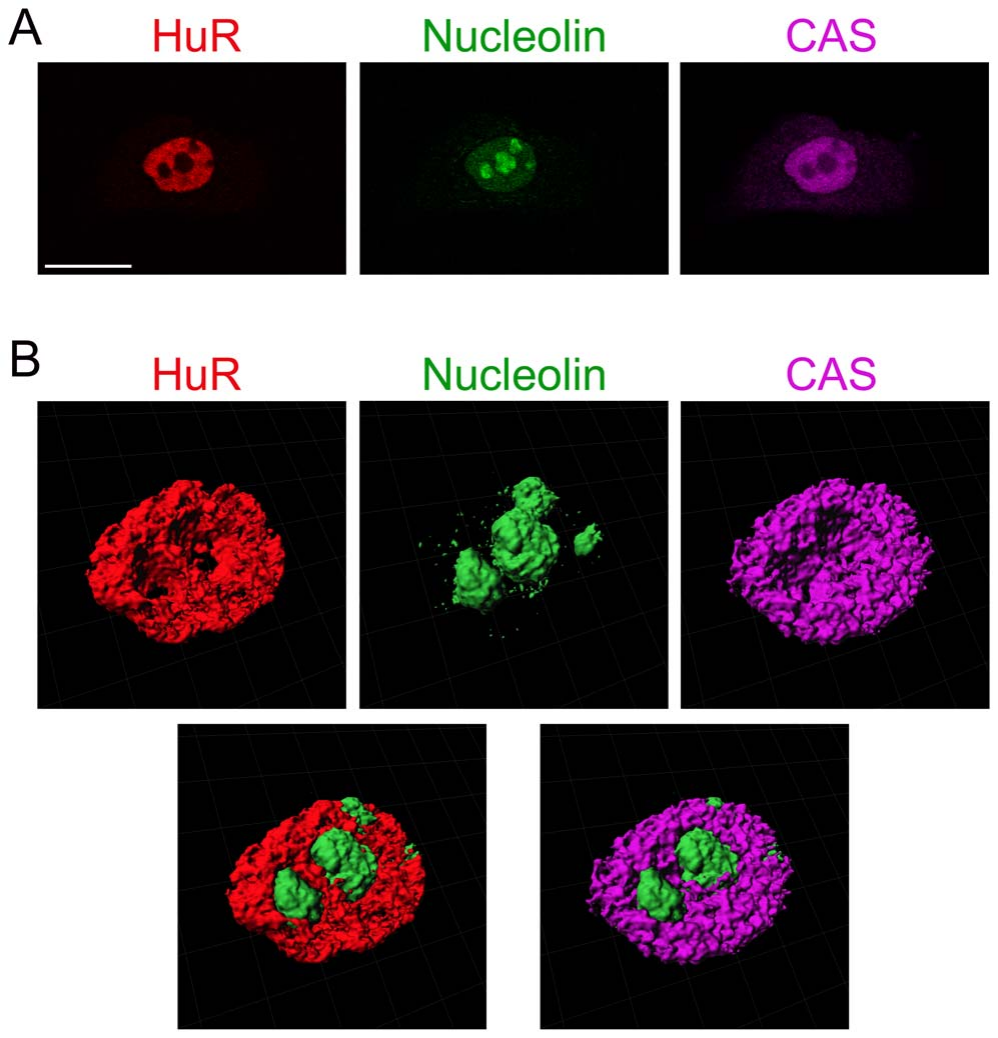
CAS, HuR and nucleolin provide appropriate references for the 3D reconstruction of nucleoli. (A) HeLa cells were stained with antibodies against, HuR, nucleolin and CAS as described for Fig. 1. A z-stack was acquired and a single slice of the stack is depicted. Size bar is 20 µm. (B) The z-stack was processed with Imaris (Bitplane) software to generate isosurfaces. Images are shown for HuR (red), nucleolin (green) and CAS (magenta). Bottom panels show the combination of either HuR and nucleolin (left) or CAS and nucleolin (right).

**Figure 11:**
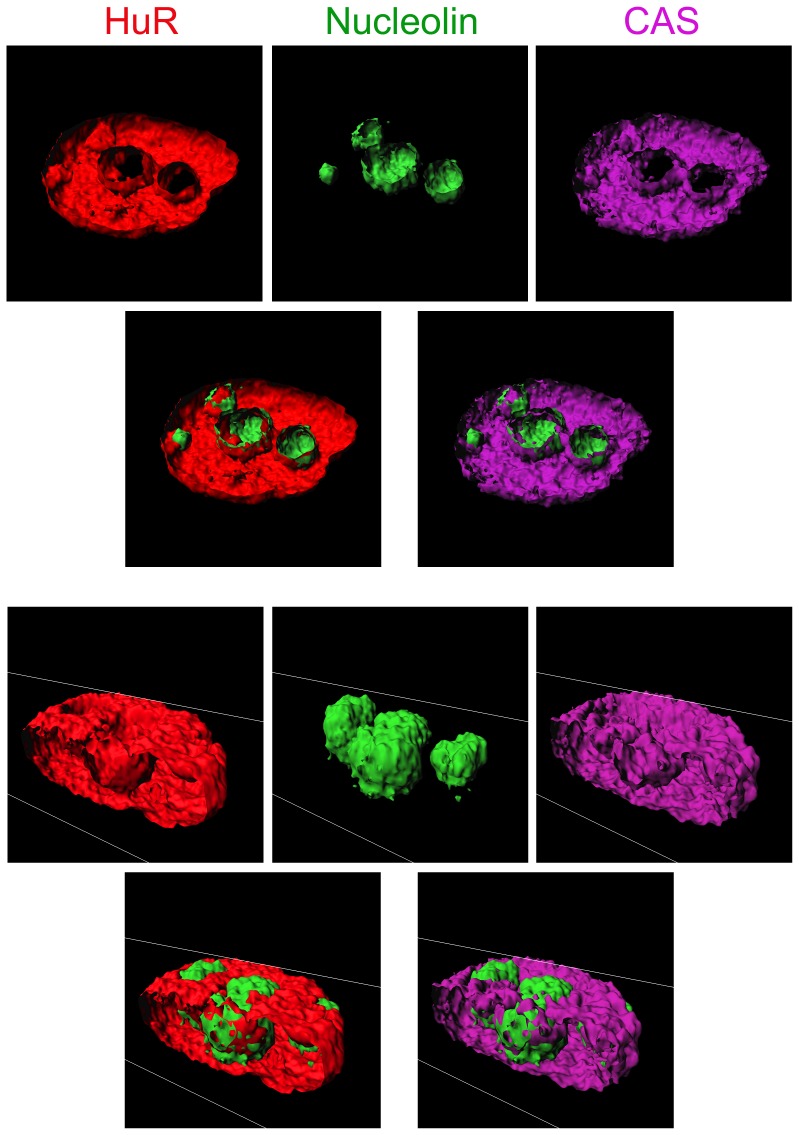
CAS, HuR and nucleolin demarcate nucleoli in 3D. Images for isosurfaces depicted in Fig. 10 were sliced in two different planes. The panels display the results for HuR (red), nucleolin (green) and CAS (magenta). Overlays show the combination of either HuR and nucleolin or CAS and nucleolin.

The results above examined the suitability of CAS, HuR and nucleolin for the nucleolar identification in optical sections. However, measurements of nucleolar volumes need to define compartment boundaries in 3D. To assess the performance of CAS, HuR and nucleolin in such applications, 3D images were reconstructed from stacks of optical sections (Fig. 10), and nuclei were sectioned in two different planes (Fig. 11). The surface rendering and sectioning demonstrated that HuR, nucleolin and CAS properly demarcated nucleoli in these images.

### Identification of nucleoli with ImageJ

ImageJ is free software that is frequently used for image analysis [44]; this software was applied to demarcate compartments for a nucleolin image (Fig. S6). To achieve this, a threshold image was created to generate masks. Masks were then overlaid with the original image to show the segmentation. ImageJ outlined the segments (blue in Fig. S6) and numbered individual nucleoli.

It is also possible to create nucleolar segments with ImageJ when a marker is excluded from nucleoli (data not shown). However, there is no fast and simple way to overlay these marker-derived segments with another image. Therefore, ImageJ is currently not efficient for the identification of nucleoli based on CAS or HuR.

## Discussion

Aside from its fundamental function in ribosome biogenesis, the nucleolus is also a key player for many other biological processes. For example, nucleoli are crucial for the stress response, cell cycle progression, apoptosis, virus replication and tumorigenesis. To quantitatively assess the role of nucleoli in cell physiology, powerful tools are necessary that measure changes in the structural and functional organization of these compartments under different growth conditions. Previously, we developed quantitative immunofluorescence methods that determine the association of fluorescent molecules with nucleoli [21]. Despite the successful application of these protocols, the nucleolar markers described earlier are not always suitable to study nucleoli. It was therefore our goal to identify novel nucleolar markers that cover a wider range of experimental settings, including those that lead to nucleolar fragmentation.

In the current contribution, we assessed three different proteins for their potential use as nucleolar markers in two different cell lines. We have tested these candidates under conditions that redistribute many of the well-characterized nucleolar proteins; i.e. heat or oxidative stress and two chemotherapeutic compounds that inhibit transcription. As both of these drugs, actinomycin D and DRB, relocate nucleolar proteins and ultimately fragment nucleoli, they provided an excellent choice to examine the robustness of our methods and the reliability of potential nucleolar markers.

Specifically, we showed that nucleolin successfully demarcated nucleoli when cells were exposed to heat, oxidant or actinomycin D. However, this did not apply to DRB treatment which required alternative markers for nucleolar demarcation. To this end, we have expanded the concept of “negative markers for nucleoli”, i.e. identification of nucleoli with factors that are excluded from this compartment. With this approach, we validated the nuclear carrier CAS and the RNA-binding protein HuR as negative markers for nucleoli; they were superior to nucleolin for treatments which severely changed the nucleolar organization, as exemplified by actinomycin D and DRB.

While CAS and HuR delimited nucleoli successfully under various experimental conditions, the accuracy of nucleolar identification was further improved by combining the information from CAS and HuR images. Consequently, this strategy detected nucleoli that were missed when each marker was employed individually. We have previously shown that some experimental settings, such as high-throughput screening, are not compatible with visual inspection during image analyses. Thus, combining multiple nucleolar markers, as described here, will improve the detection process and eliminate the need for visual inspection [21].

Our validation of CAS and HuR as novel nucleolar markers sets the stage to analyze in a quantitative fashion how the diverse array of biological processes located in nucleoli is altered by physiological changes or pharmacological intervention. As the nucleolus responds to stress and chemotherapeutic agents by redistributing many of its resident proteins, “negative” markers that are excluded from nucleoli offer an ideal tool to delimit this compartment. Indeed, we have applied these tools to define how B23 and nucleolin respond to DEM and DRB. While the oxidant DEM did not change the nucleolar concentration of B23, a small but significant increase took place for nucleolin. Future experiments will have to determine the mechanisms underlying this reaction. For example, DEM could stimulate the synthesis, prevent the degradation or relocate nucleolin within the cell. In contrast to DEM, DRB had a similar effect on B23 and nucleolin, as the agent reduced the nucleolar concentration of both proteins to the same extent.

We obtained further validation for our protocols by quantifying the stress and drug-dependent inhibition of nucleolar function. The measurements of *de novo* synthesized RNA in nucleoli revealed that DEM, actinomycin D and DRB caused a pronounced inhibition of RNA synthesis in nucleoli. At the same time, all of the compounds reorganized nucleoli, as they altered the concentration of B23 and/or nucleolin. Collectively, these results give additional credit to the idea that the function of the nucleolus is controlled by its organization [2].

Since there is no universal marker to identify nucleoli under all conditions, our research provides a road map for the selection of markers that are appropriate for specific settings. On the basis of our results, Table 1 recommends such makers for the conditions tested by us. We anticipate that this information will build the foundation for a database of nucleolar marker proteins that are suitable for diverse experimental settings.

**Table 1:** Markers that identify nucleoli under various experimental conditions.

Treatment	Markers for the identification of nucleoli		
	Nucleolin	CAS	HuR
Heat shock and recovery	Excellent	Poor	Poor
Oxidative stress	Excellent	Good	Good
Actinomycin D	Excellent	Excellent	Excellent
DRB	Poor	Good^1^	Good^1^

Nucleolin, CAS and HuR were assessed with the software operations described in Materials and Methods. Specifically, we evaluated the ability of each candidate to generate segments that colocalize with black or bright holes, respectively. This assessment was performed for the different experimental conditions listed.

1 For DRB treatment, the most accurate demarcation of nucleoli was obtained when CAS and HuR were combined as markers.

## Supporting Information

Figure S1 **Western blot analysis monitors the specificity of antibodies used to demarcate nucleoli.** Crude extracts were prepared for HeLa and MCF7 cells, and Western blots were incubated with antibodies against CAS, HuR, nucleolin or nucleostemin. Adjacent lanes of the same filter were probed with isotype-specific control antibodies (IgG). Control IgGs were used at the same concentration as primary antibodies and exposure times for enhanced chemiluminescence was identical for primary and control antibodies.
(TIF)

Figure S2 **Validation of antibodies used for the immunolocalization of CAS, HuR, nucleolin and nucleostemin.** HeLa or MCF7 cells were processed for immunostaining with antibodies against CAS, HuR, nucleolin or nucleostemin as described in Materials and Methods. Each antigen was detected with fluorescent secondary antibodies against goat (CAS, nucleostemin), mouse (HuR) or rabbit (nucleolin). In control experiments, primary antibodies were omitted (no primary), and samples were incubated with a combination of the three secondary antibodies. For each cell line, all of the images were acquired with identical settings of the microscope. Fluorescence signals are shown for DAPI and three additional channels: channel 1, far red; channel 2, red; channel 3, green emission. Size bar is 20 µm.
(TIF)

Figure S3 **Immunolocalization of nucleolin and nucleostemin.** HeLa and MCF7 cells were stained simultaneously with antibodies against nucleolin and nucleostemin. Two different images are shown for each cell type. Fluorescence signals are magenta for nucleostemin and green for nucleolin. Overlap of the signals was detected by merging the images for both proteins. A 300X magnified view depicts the overlay images for several nucleoli. Size bar is 20 µm.
(TIF)

Figure S4 **Detection of nucleoli with HP1γ.** HeLa cells were stained with antibodies against CAS, HP1γ and nucleolin (NCL). Panels depict single staining or different overlays as indicated in the figure. Size bar is 20 µm.
(TIF)

Figure S5 **CAS, HuR and nucleolin delimit the nucleolus after treatment with low concentrations of actinomycin D.** HeLa cells were incubated with the vehicle DMSO, 1 nM or 10 nM actinomycin D for 6 hours and stained with antibodies against CAS, HuR and nucleolin. Nucleolar detection and segments overlay was performed as described for 100 nM actinomycin D in Fig. 3. Size bar is 20 µm. Note that after treatment with 1 nM or 10 nM actinomycin D, CAS, HuR and nucleolin are suitable markers to demarcate nucleoli with the “detect dark holes” filter.
(TIF)

Figure S6 **Detection of nucleoli with ImageJ.** Nucleoli were identified with ImageJ, beginning with an original nucleolin image in tif-format. Thresholding was carried out with the *Adjust* tab and *Threshold* command. The resulting Threshold image was used to create a Mask image by following the *EditSelectionCreate Mask* command. Pixel values were then selected in the *AnalyzeAnalyze Particles* option. The segmentation defined nucleolar compartments, which were outlined and numbered. A selected region of the segmentation image was magnified 500X to display the outline and numbering. Fluorescent intensities can be measured in nucleolar compartments with the *AnalyzeMeasure* command (not shown).
(TIF)
