# Supplementary figures and images for: Identification of Novel Markers That Demarcate the Nucleolus during Severe Stress and Chemotherapeutic Treatment
Source: PLoS One. 2013 Nov 6;8(11):e80237. doi: 10.1371/journal.pone.0080237 (PMC3819286; doi:10.1371/journal.pone.0080237)

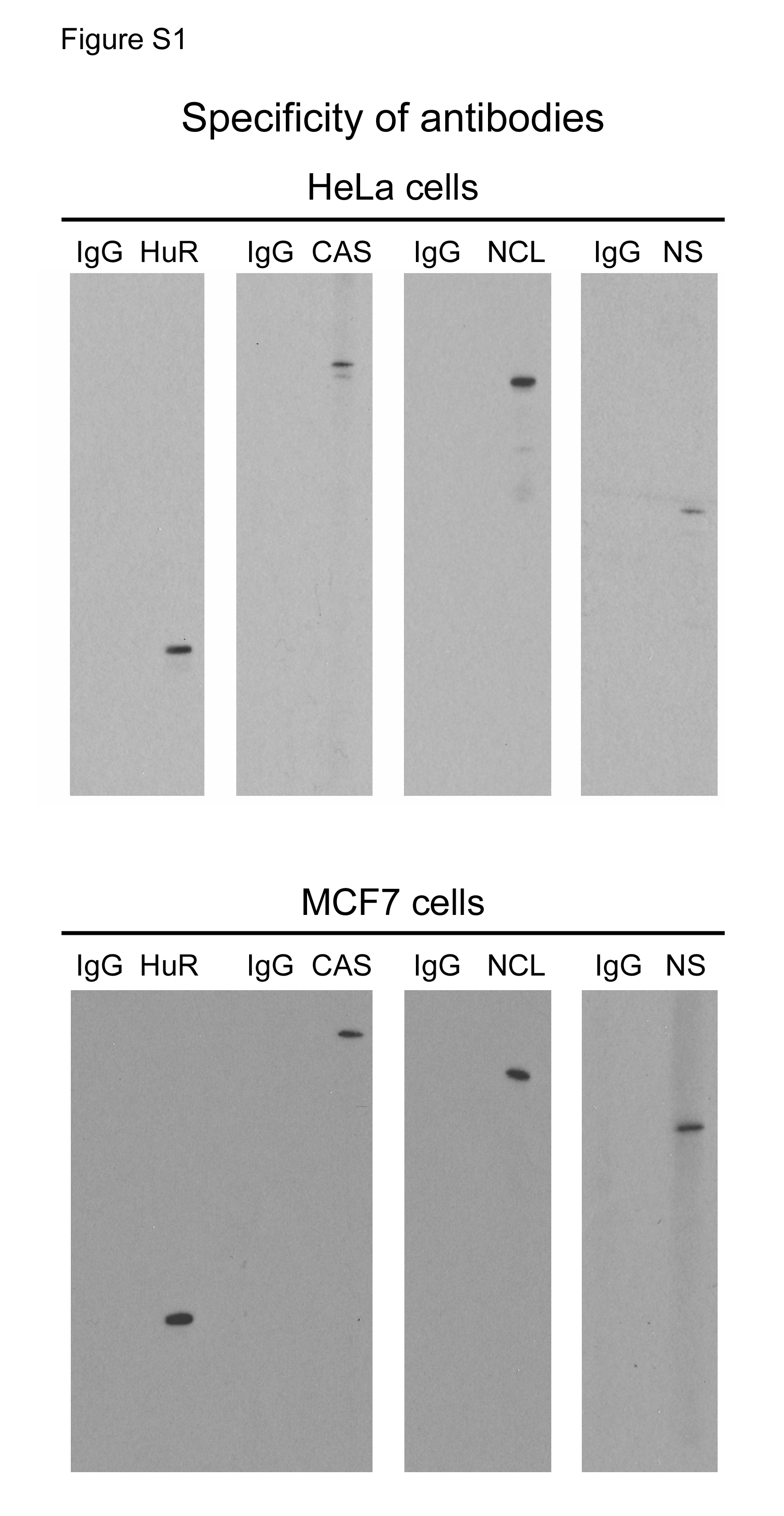

Supplement: Figure S1 — Western blot analysis monitors the specificity of antibodies used to demarcate nucleoli. Crude extracts were prepared for HeLa and MCF7 cells, and Western blots were incubated with antibodies against CAS, HuR, nucleolin or nucleostemin. Adjacent lanes of the same filter were probed with isotype-specific control antibodies (IgG). Control IgGs were used at the same concentration as primary antibodies and exposure times for enhanced chemiluminescence was identical for primary and control antibodies. (TIF) [file pone.0080237.s001.tif]

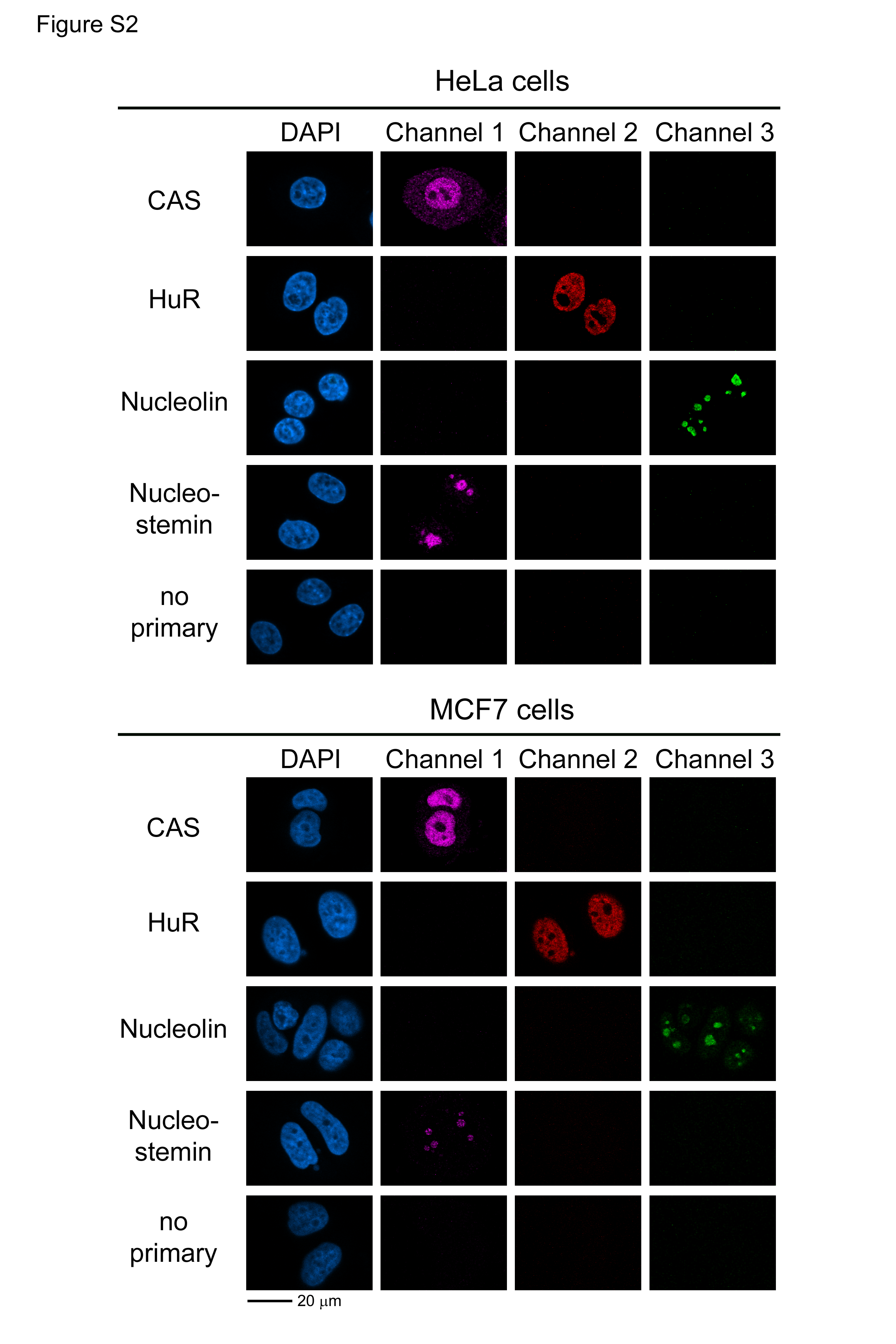

Supplement: Figure S2 — Validation of antibodies used for the immunolocalization of CAS, HuR, nucleolin and nucleostemin. HeLa or MCF7 cells were processed for immunostaining with antibodies against CAS, HuR, nucleolin or nucleostemin as described in Materials and Methods. Each antigen was detected with fluorescent secondary antibodies against goat (CAS, nucleostemin), mouse (HuR) or rabbit (nucleolin). In control experiments, primary antibodies were omitted (no primary), and samples were incubated with a combination of the three secondary antibodies. For each cell line, all of the images were acquired with identical settings of the microscope. Fluorescence signals are shown for DAPI and three additional channels: channel 1, far red; channel 2, red; channel 3, green emission. Size bar is 20 µm. (TIF) [file pone.0080237.s002.tif]

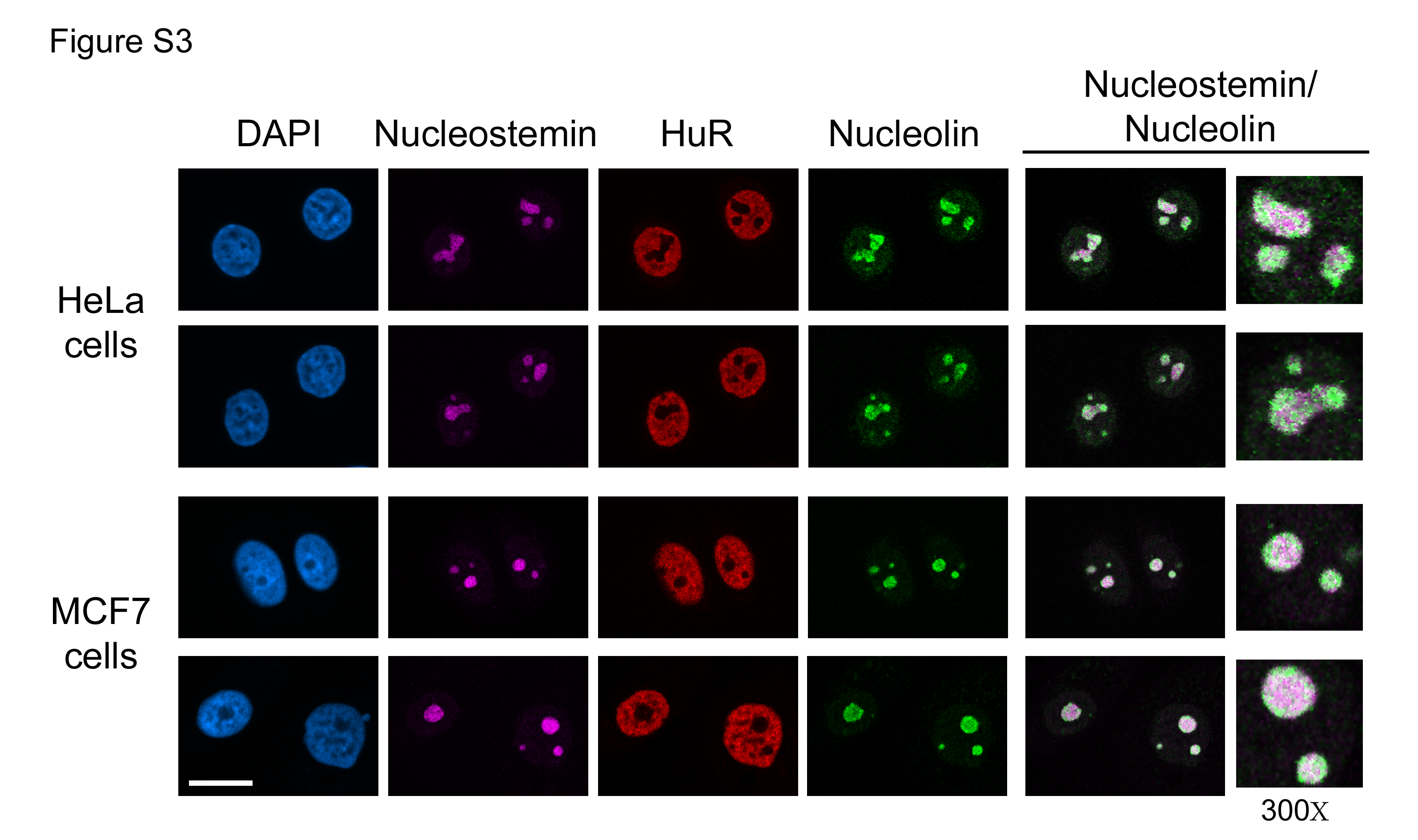

Supplement: Figure S3 — Immunolocalization of nucleolin and nucleostemin. HeLa and MCF7 cells were stained simultaneously with antibodies against nucleolin and nucleostemin. Two different images are shown for each cell type. Fluorescence signals are magenta for nucleostemin and green for nucleolin. Overlap of the signals was detected by merging the images for both proteins. A 300X magnified view depicts the overlay images for several nucleoli. Size bar is 20 µm. (TIF) [file pone.0080237.s003.tif]

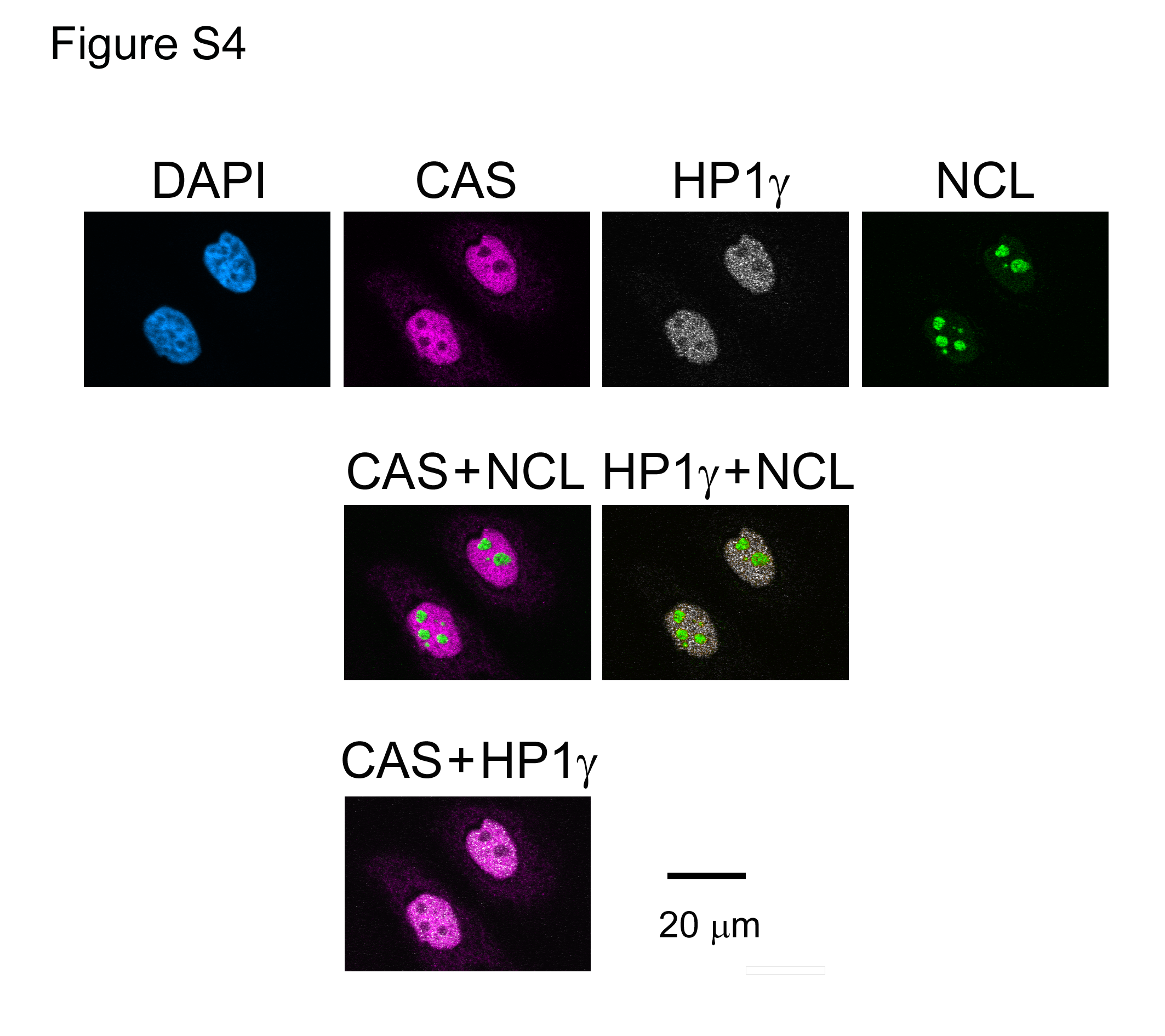

Supplement: Figure S4 — Detection of nucleoli with HP1γ. HeLa cells were stained with antibodies against CAS, HP1γ and nucleolin (NCL). Panels depict single staining or different overlays as indicated in the figure. Size bar is 20 µm. (TIF) [file pone.0080237.s004.tif]

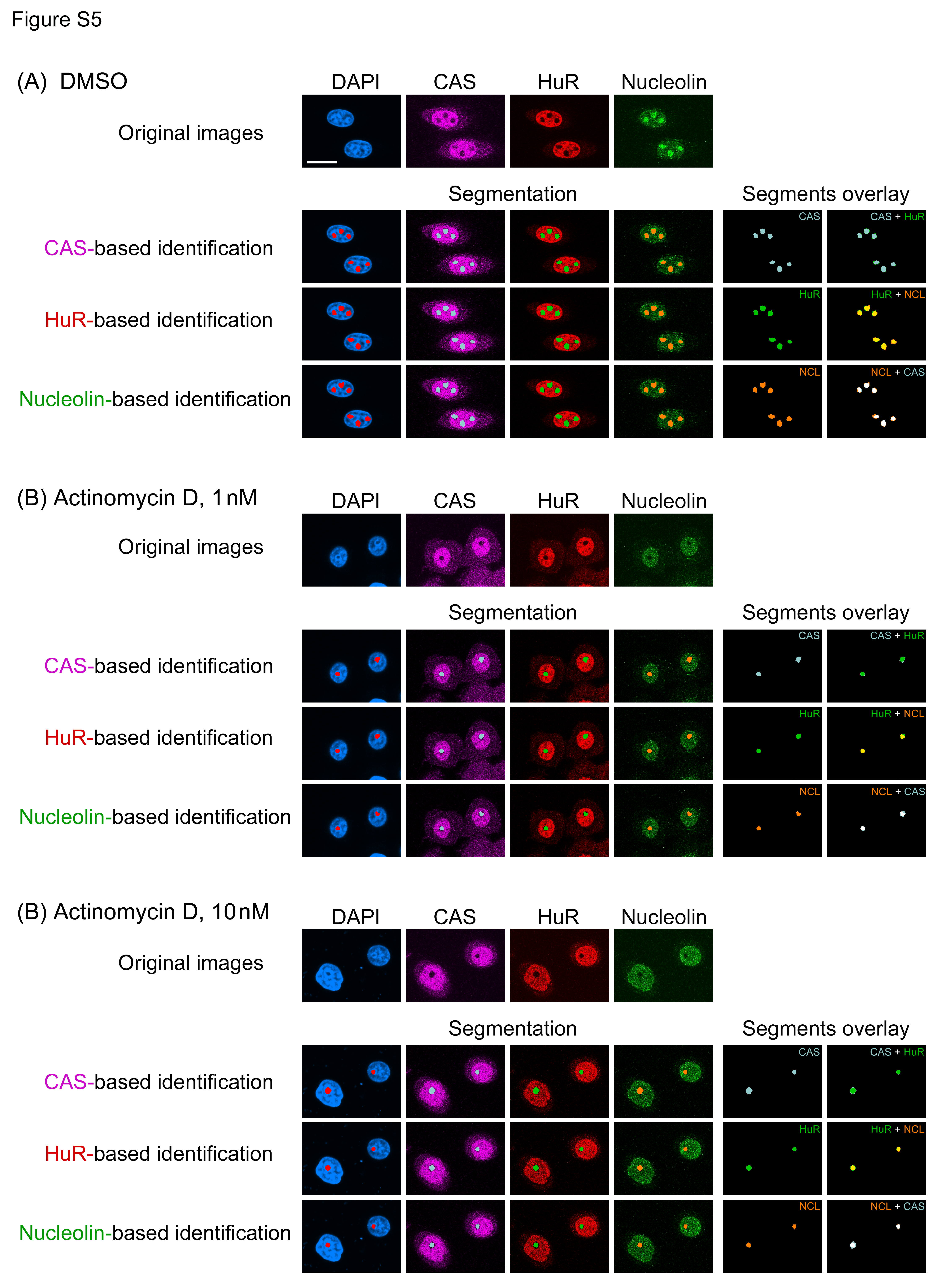

Supplement: Figure S5 — CAS, HuR and nucleolin delimit the nucleolus after treatment with low concentrations of actinomycin D. HeLa cells were incubated with the vehicle DMSO, 1 nM or 10 nM actinomycin D for 6 hours and stained with antibodies against CAS, HuR and nucleolin. Nucleolar detection and segments overlay was performed as described for 100 nM actinomycin D in Fig. 3. Size bar is 20 µm. Note that after treatment with 1 nM or 10 nM actinomycin D, CAS, HuR and nucleolin are suitable markers to demarcate nucleoli with the “detect dark holes” filter. (TIF) [file pone.0080237.s005.tif]

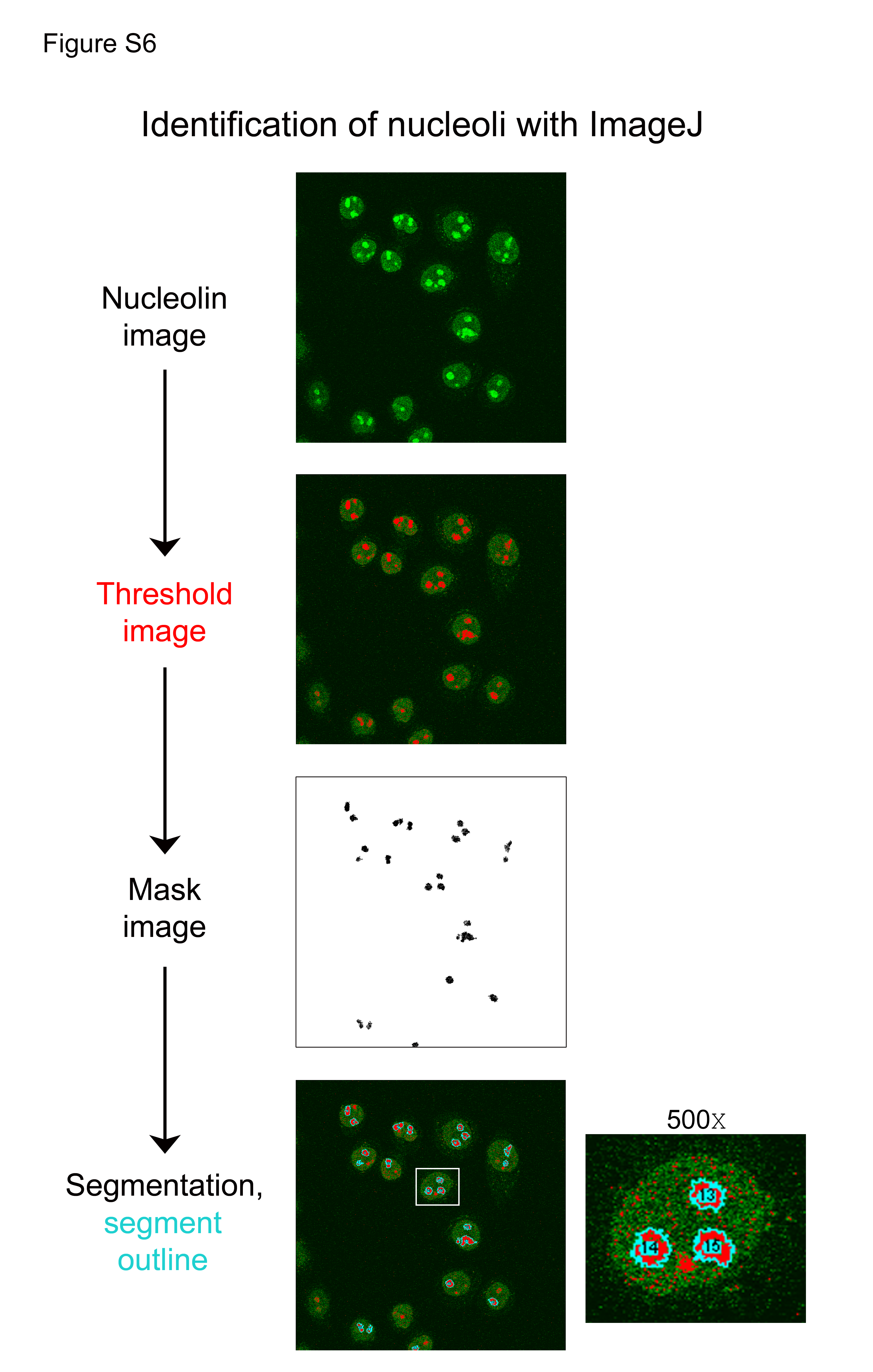

Supplement: Figure S6 — Detection of nucleoli with ImageJ. Nucleoli were identified with ImageJ, beginning with an original nucleolin image in tif-format. Thresholding was carried out with the Adjust tab and Threshold command. The resulting Threshold image was used to create a Mask image by following the EditSelectionCreate Mask command. Pixel values were then selected in the AnalyzeAnalyze Particles option. The segmentation defined nucleolar compartments, which were outlined and numbered. A selected region of the segmentation image was magnified 500X to display the outline and numbering. Fluorescent intensities can be measured in nucleolar compartments with the AnalyzeMeasure command (not shown). (TIF) [file pone.0080237.s006.tif]
